# Vertically Transferred Immunity in Neonates: Mothers, Mechanisms and Mediators

**DOI:** 10.3389/fimmu.2020.00555

**Published:** 2020-03-31

**Authors:** Marie Albrecht, Petra Clara Arck

**Affiliations:** Laboratory for Experimental Feto-Maternal Medicine, Department of Gynecology and Obstetrics, University Medical Center Hamburg-Eppendorf, Hamburg, Germany

**Keywords:** maternal vaccination, measles, rubella, pertussis, influenza, FcRn, blunting, breastfeeding

## Abstract

Over the last years, an increasing number of outbreaks of vaccine-preventable infectious diseases has been reported. Besides elderly and immunocompromised individuals, newborns and small infants are most susceptible to infections, as their immune system is still immature. This vulnerability during infancy can be mitigated by the transplacental transfer of pathogen-specific antibodies and other mediators of immunity from mother to the fetus during pregnancy, followed postnatally by breast milk-derived immunity. Since this largely antibody-mediated passive immunity can prevent the newborn from infections, neonatal immunity depends strongly on the maternal concentration of respective specific antibodies during pregnancy. If titers are low or wane rapidly after birth, the protection transferred to the child may not be sufficient to prevent disease. Moreover, emerging concepts propose that mothers may transfer active immunity to the newborns via vertical transfer of pathogen-specific T cells. Overall, a promising strategy to augment and prolong neonatal immunity is to vaccinate the mother before or during pregnancy in order to boost maternal antibody concentrations or availability of specific T cells. Hence, a large number of pre-and postconceptional vaccine trials have been carried out to test and confirm this concept. We here highlight novel insights arising from recent research endeavors on the influence of prenatal maternal vaccination against pathogens that can pose a threat for newborns, such as measles, pertussis, rubella and influenza A. We delineate pathways involved in the transfer of specific maternal antibodies. We also discuss the consequences for children’s health and long-term immunity resulting from an adjustment of prenatal vaccination regimes.

## Early Life Immunity and Time Windows Permitting Pathogen Threats for Neonates

After birth and during their first months of life, human newborns are not yet equipped with a fully matured immune system (1, 2). Hence, they are highly susceptible to infectious pathogens, such as measles, pertussis, rubella, and influenza. These pathogens can cause a severe course of disease in neonates and infants, which may even be fatal (3–5). The availability of safe and immunogenic vaccines against infectious diseases, i.e., the combined measles-mumps and rubella vaccine, does not mitigate this threat to neonatal health, as the vaccines contain living pathogen components; hence, their use is not recommended to be administered to children under the age of 12 months. Similarly, the vaccination with the combined tetanus-diphtheria-pertussis (Tdap) vaccine and the inactivated influenza vaccines (IIV) is not recommended until 2 or 6 months of age, respectively (6, 7). These restrictions to vaccination leave a pivotal gap of neonatal immunity against these pathogens until routine immunization can be administered (8).

This gap in immunity is – at least in part – covered by the active, transplacental transfer of maternal pathogen-specific antibodies. Mothers convey passive immunity to their newborns through the transplacental transfer of antibodies, hereby providing a shield for the infant from pathogen-mediated diseases (1, 9). The amount of transferred antibodies can differ between individuals and is mainly dependent on maternal antibody concentrations (10, 11). Based on this natural immunity mediated by the mother, maternal vaccination strategies during pregnancy are vividly discussed. Such strategies could increase maternal antibody concentrations, enhance the levels of transplacental antibody transfer and, in consequence, the degree of passive immunity for the neonate (12).

**FIGURE 1:**
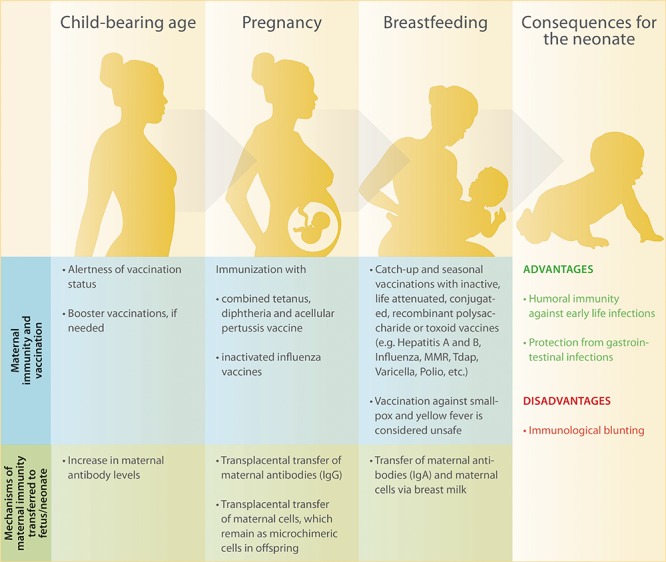
Overview of maternal immunity and recommended vaccinations before, during and after pregnancy as well as consequences for maternal and children’s health.

In the light of the recent outbreaks of vaccine-preventable diseases such as measles even in countries with high vaccine coverage, the topic of immunization has received significant attention by medical professionals and the lay community. Measles infection has caused more than 140,000 deaths globally in 2018, most of them among children under five years of age (13). Promoting the immunity of newborns via maternal vaccination holds the potential to become an effective and low-cost approach to prevent neonatal morbidity and mortality caused by communicable diseases (14–16). In the present article, we comprehensively discuss recent research studies on maternal vaccination against common childhood infections such as pertussis, influenza, measles, and rubella. We further highlight pathways involved in the transplacental transfer of antibodies as well as mechanisms through which neonatal immunity can be improved irrespective of maternal antibodies (Figure 1).

## Observations From Vaccination Studies Against Tetanus, Diphtheria and Pertussis During Pregnancy

A number of recent studies confirm that vaccination with the combined tetanus, diphtheria, and acellular pertussis vaccine (Tdap) can be recommended during pregnancy, since vaccine trials carried out on a large scale and in various countries have generally demonstrated its safety and immunogenicity in mothers and their infants (Table 1). The World Health Organization (WHO) reports a 96% reduction of death by neonatal tetanus through implementation of recommended elimination practices from 1988 to 2015, including the vaccination of pregnant women (17). Similarly, the burden of diphtheria disease has been reduced (18). Unfortunately, comparable achievements have not been made with regard to pertussis elimination. Outbreaks of whooping cough have recently been occurring worldwide, exposing young infants to a particularly high risk of severe infections. Thus, we here mainly discuss studies that focus on the outcome of pertussis vaccination in pregnant women.

**TABLE 1:** Overview of studies and trials assessing safety, effectiveness and outcome of vaccinations with Tdap, IIV, and MMR during child-bearing years, pregnancy or infancy in humans.

**Aim of study**	**Study design**	***N***	**References**
**Pathogens: *C. tetani, C. diphtheriae*, and *B. pertussis***			
Assessment of immunity against vaccine preventable diseases	Prospective, observational study	194	(1)
Safety and immunogenicity of Tdap matVac, interference of matAB	Randomized double-blind controlled clinical trial	171	(19)
Effect of 2 doses of pertussis vaccine before 2 months of age	Randomized non-blinded clinical trial	76	(22)
Assessment of B. pertussis titers in third trimester and newborns	Observational, cross-sectional study	111	(23)
Maternal outcome upon Tdap matVac	Retrospective observational study	68,550	(24)
Assess effectiveness of Tdap matVac	Matched case-control study	234	(28)
VE in protecting newborns from pertussis infection	Matched case-control study	88	(29)
Comparative analysis of Tdap matVac timepoint and AB yield in newborn	Prospective study	81	(30)
Determination of optimal GW for Tdap matVac in third trimester	Prospective study	154	(31)
Comparative analysis of Tdap matVac in second or third trimester	Prospective observational study	335	(32)
Effect of Tdap booster dose between two pregnancies	Prospective study	144	(72)
Comparative analysis of maternal and cord blood AB and proteins at term	Observational study	16	(73)
Analysis of neutralizing antibodies in infants after vaccination against diphtheria	Prospective study	44	(94)
Effect of matVac with Tdap and IIV on infant AB responses	Prospective study	369	(95)
Influence of Tdap booster dose during pregnancy on infant’s matAB levels and immune responses	Prospective controlled cohort study	99	(96)
Safety and immunogenicity of Tdap matVAc and effect on infant immune responses	Randomized, double-blind, placebo-controlled trial	80	(97)
**Pathogen: Influenza A**			
Assessment of safety and immunogenicity of seasonal trivalent IIV matVac	Prospective, randomized, double-blind clinical trial	100	(40)
Risk assessment for neonatal birth defects after first trimester IIV exposure	Observational study	425,944	(41)
Persistence of HAI titers and VE of IIV3 in subsequent influenza season in women	Double-blind, randomized, placebo-controlled trial	479	(43)
Duration of infant protection upon IIV matVac	Substudy of randomized, double-blind, placebo-controlled clinical trial	322	(44)
Clinical effectiveness of IIV matVac; safety and immunogenicity of pneumococcal vaccines	Prospective, controlled, blinded, randomized study	340	(45)
Risk assessment for infant hospitalization due to lower respiratory infection after IIV matVac	Secondary analysis of randomized controlled trial	52	(46)
Effect of IIV matVac on risk for influenza in infants < 6 months of age	Non-randomized, prospective, observational cohort study	1169	(47)
Influence of IIV matVac on subsequent B. pertussis infection rates in mothers	Retrospective testing of samples collected in randomized controlled trial	3125	(48)
Effect of vitamin A supplementation on immune response to IIV matVac	Prospective study	112	(70)
Investigation of sensitization to IIV antigens *in utero*	Prospective observational study	126	(74)
Effect of maternal influenza vaccination on influenza-specific IgA levels in breast milk	Prospective, blinded, controlled trial	340	(80)
Effect of cross-reactive cellular immunity on symptomatic influenza illness in AB- naïve individuals	Prospective study	342	(90)
**Pathogens: Measles, Mumps, and Rubella Virus**			
Repertoire of maternal anti-viral AB in newborns at birth	Prospective study	78	(10)
Assessment of safety of MMR vaccination in adults	Retrospective observational study	3175	(51)
Assessment of B cell impairment upon measles-associated immunosuppression	Prospective observational study	29	(54)
Identification of measles infection long- term effects on immune system	Prospective study	196	(55)
Association of maternal age and vaccination status with cord blood matAB	Observational study	206	(57)
MatAB transfer in vaccinated or naturally immune mothers to preterm/term infants	Prospective study	195	(58)
Quantification of AB against MMR and varicella zoster in mothers and infants	Prospective observational study	138	(59)
Duration of presence of matAB to measles in infants	Prospective study	207	(60)
Seronegativity in infants < 6 months and serologic response to measles vaccine	Cross-sectional study	203	(61)
Prenatal fetal infection among women (re-) infected with rubella during pregnancy	Prospective observational study	40	(62)
Detection of rubella-specific IgM in subclinical rubella reinfection in pregnancy	Case report	8	(63)
Criteria for defining rubella reinfection	Case report	5	(64)
Fetal infection after maternal rubella reinfection during pregnancy	Case report	1	(65)
Seroepidemiology of anti-measles, -mumps and -rubella AB in pregnant women and neonates	Prospective study	353	(71)
Assessment of transplacental transport of IgG immune complexes	Prospective study	152	(75)
Immunogenicity of measles vaccine in infants < 12 months	Cohort study	72	(92)
**Studies on breast milk immunity**			
Assessment of gut microbiota bound by breast milk IgA	Observational study	69	(81)
Effects of infections during pregnancy on colostrum IgA levels	Cross- sectional study	900	(82)

*matVac, maternal vaccination during pregnancy; Tdap, Tetanus, diphtheria, acellular pertussis vaccine; matAB, maternal antibody; VE, vaccine effectiveness; GW, gestational week; AB, antibody; IIV(3), (trivalent) inactivated influenza vaccine; MMR, measles, mumps, rubella vaccine; HAI, hemagglutination inhibition assay.*

Amongst others, the authors of a recent study aimed to evaluate the safety and immunogenicity of Tdap administration during pregnancy in mothers and their infants and to assess the possible interference of maternal antibodies with subsequent infant immunizations (19). Apart from mild and self-limiting local reactions at the vaccination site, no adverse events caused by the immunization with Tdap were reported in mothers and their infants. Anti-pertussis toxin (PT) antibodies, which primarily mediate protection against Bordetella pertussis-induced disease (20), and anti-pertactin (PRN) antibodies, which convey protection by opsonization and subsequent phagocytosis of Bordetella pertussis (21), were significantly increased in mothers vaccinated with Tdap during pregnancy, compared to the placebo group. Accordingly, both anti-PT and anti-PRN were significantly higher at birth in infants of vaccinated mothers. Irrespective of prenatal vaccination, cord blood antibody titers exceeded maternal titers assessed at delivery, indicating an active transplacental transport of antibody. However, anti-PT and anti-PRN decreased quickly until the age of 2 months.

The investigators also pointed out differences in anti-PRN and anti-PT seroresponses following routine infant vaccinations at 2 and 4 months of age with a combined tetanus, diphtheria, pertussis, polio and Hib vaccine (19). After vaccination, infants of placebo-receiving mothers showed a greater increase of anti-PT levels compared to infants of Tdap-vaccinated mothers, indicating an interference of maternal antibodies with the child’s seroresponse to vaccination. Surprisingly, opposed to the response to PT, an anti-PRN response was not mounted in these infants, irrespective of maternal Tdap vaccination. This is in contrast to a study focusing on infants’ response to Tdap vaccination during early life, in which a significant seroresponse to both PT and PRN was mounted (22). An explanation for the ambiguity between the vaccination responses observed in these two studies cannot be deduced from the respective articles, but may be due to different cohort sizes, variations in the procedure of specimen preparation or the different ELISA kits used to determine antibody concentrations.

Another study focusing on the influence of maternal vaccination with Tdap during the second trimester of pregnancy (23) revealed that anti-PT IgG could be detected in 92% of infants born to vaccinated mothers, whilst anti-PT IgG was undetectable in infants of unvaccinated mothers. Although this study has some limitations, for example the lack of initial maternal anti-PT levels and the ELISA-based analysis allowing for detection of antibody presence or absence only, but no concentrations, it shows that maternal immunization with Tdap during the 2nd trimester of pregnancy significantly increases the percentage of seropositive newborns.

Not only immune responses of mother and child toward Tdap immunization during pregnancy have been investigated, but also vaccine safety. By using information from different national databases, Griffin et al. (24) identified a large cohort of women who were eligible to receive governmental funded Tdap vaccination between gestational week 28 and 38. Hospitalization for severe pregnancy complications was set as the primary outcome and hospitalizations for less critical pregnancy complications as secondary outcomes. Key finding of this study was that the hazard ratio for primary or secondary outcomes did not increase when Tdap was administered during pregnancy. Intriguingly, the authors also report that Tdap vaccination during pregnancy significantly reduced the risk for hospitalization due to severe pre-eclampsia, as well as the risk for antenatal bleeding and preterm labor and delivery. Upon inspection of the studied population, these risk reductions might be biased by the demographic characteristics that distinguish vaccinated and unvaccinated women. Vaccinated women tended to be European, have a higher income level and receive care from an obstetrician. Since pregnancy complications as well as mother and infant mortality are rather associated with lower socioeconomic status and non-caucasian ethnicity (25–27), it is tempting to assume that higher rates for primary and secondary outcomes observed in this study may be due to confounders.

Noteworthy, New Zealand had been facing a large pertussis epidemic from 2011 to 2013. However, only 11.9% of the individuals eligible to receive Tdap in the study by Griffin et al. have been vaccinated. This example shows the urgent need for further education of the population regarding the effectiveness of immunization against pertussis.

Whilst the evidence for safety and immunogenicity of Tdap is steadily increasing, Saul et al. also emphasized on the effectiveness of maternal Tdap vaccination with regard to infant hospitalization due to pertussis infection (28). The authors report a 39% vaccine effectiveness (VE) to prevent pertussis infection for infants < 6 months and of 69% for infants younger than 3 months of age; the overall VE against hospitalization due to severe pertussis infection was 94%. These results clearly demonstrate that maternal Tdap vaccination is predominantly effective in preventing severe cases of pertussis disease, with maternal vaccination attenuating the intensity of the illness rather than preventing it. Furthermore, the authors identified that breastfeeding may have a protective effect on pertussis infection of the infant. These findings are in line with a very similarly set up of a study conducted in the same year (29). Here, the authors found a 90.9% VE of maternal Tdap vaccination during pregnancy in protecting infants < 3 months from laboratory confirmed pertussis; yet, VE was calculated from a small cohort. Also, apart from maternal vaccination, breastfeeding was identified as the only other significant influence on infant protection against pertussis. This effect could be observed not only in mothers vaccinated during pregnancy, where maternal IgA could be passed via the breast milk, but also in those who had not been vaccinated against or in contact with pertussis for the last 10 years. The authors suggest that this might be attributed to other breast milk components which were not further specified.

There is still ambiguity with regard to vaccination timepoint recommendation by national health services. The National Health Service (NHS) in the United Kingdom and the Advisory Committee on Immunization Practices (ACIP) in the US suggest two different vaccination schedules. While the NHS recommends Tdap administration between 16 and 32 weeks of gestation (30), the ACIP proposes that Tdap should be administered at a later timepoint between 27 and 36 weeks of gestation (6). Using cord blood concentrations of pertussis-specific IgG as a read out parameter, one study reports highest levels if mothers had been vaccinated with Tdap between 27 and <31 weeks of gestation, as compared to vaccination at 31 weeks or later (31). Another study suggested that the optimal timepoint for Tdap administration is between 28 and 32 weeks of gestation, based on higher cord blood anti-pertussis antibody concentrations resulting from vaccination at this timepoint as compared to later in gestation (32). Conversely, another study with a higher number of participants reports that maternal Tdap vaccination between gestational week 13 and 25 results in higher cord blood anti-pertussis antibody concentrations than immunization after 26 weeks of gestation (33). A longer period of time between vaccination and childbirth allows for a greater transfer window, which may explain the observed higher cord blood titers. Re-scheduling the recommended vaccination to an earlier timepoint during pregnancy might therefore be beneficial, not only for preterm neonates (33).

Taken together, Tdap immunization should be recommended to each pregnant woman in every pregnancy, regardless of the previous vaccination status. This will yield to high maternal antibody concentrations toward the end of pregnancy, so that antibodies can be transferred at greater extent to the fetus. Whilst vaccination of the mother during the 2nd or 3rd trimester of pregnancy is safe and efficacious, the best strategy to ensure high neonatal anti-pertussis antibody concentrations seems to be vaccination between gestational week 13 and 25. Besides maternal vaccination, passive protection of the neonate via reduction of pathogen exposure can result from a so-called cocooning effect, achieved by vaccination of family members and caregivers of the newborn (34, 35). By combining these protective techniques, the risk for pertussis infection during the first months of life, until the neonate has mounted humoral and cellular immunity against this pathogen, can be reduced.

## Insights From Vaccination Studies Against Influenza During Pregnancy

Apart from Tdap, vaccination against influenza using inactivated influenza vaccines (IIV) is the only other recommended vaccination during pregnancy. Pregnant women are at high risk for severe influenza disease outcomes due to a multi-faceted failure to mount an anti-viral response. As shown in basic science approaches, this less stringent selective environment can promote the emergence of mutated influenza variants which mediate increased viral pathogenicity (36). The Robert Koch Institute, the governmental central scientific institution safeguarding public health such as the surveillance and prevention of infectious diseases in Germany, recommends vaccination against influenza for all pregnant women during the second and third trimester. For women with increased morbidity risk or preexisting medical conditions, vaccination is even recommended during the first trimester (37). Similar recommendations have been made by the ACIP in the United States (38), where vaccination against influenza is recommend at any time during normally progressing pregnancy.

These recommendations result from a wealth of studies carried out worldwide on safety, immunogenicity and efficacy of influenza vaccination during pregnancy. These studies have clearly demonstrated the advantages of protecting mother and infant from influenza disease, as extensively reviewed elsewhere (14, 15, 39).

Moreover, independent studies (Table 1) have assessed the impact of influenza vaccination on pregnancy outcomes and confirmed that the risk for structural birth defects or pregnancy complications is unaffected by maternal vaccination against influenza (40, 41). On the contrary, the frequency of infants born small for gestational age was lower among vaccinated women and the overall birth weight was higher (42). Immunogenicity analyses using hemagglutination inhibition assay (HAI) revealed that the overall reactogenicity to the inactivated influenza virus vaccine was similar between non-pregnant and pregnant individuals (40). Here, it was also reported that higher maternal age negatively correlates with seroconversion and -protection, whilst data supporting this observation have not been shown. However, another study showing that HAI titers were likely to remain elevated one year after immunization especially in women younger than 25 years of age supports the link between maternal age and immunogenicity to IIV (43).

Besides the maternal response to influenza vaccination during pregnancy, the subsequent children’s outcome upon maternal vaccination has also been the focus of a number of studies. Here, an overall beneficial response could be identified, such as a lower hospitalization rate and milder disease course of infants < 6 months, not only related to influenza infection (44, 45), but also to all-cause lower respiratory tract infection (ALRI), including diseases induced by pathogens such as B. pertussis, respiratory syncytial virus (RSV) and rhinovirus (42, 46, 47). This broad protection from lower respiratory tract infections has been explained by an increased susceptibility to pathogens affecting the airway system subsequent to an influenza infection, from which neonates with maternally inherited passive immunity against influenza are protected to a higher degree (46, 48). However, large-scale studies are urgently needed to confirm this suggestion. Once confirmed, such insights will likely increase the vaccination compliance of pregnant women, which is still surprisingly low (49).

## Vaccines Contraindicated for Immunization During Pregnancy

Unlike vaccinations against tetanus, diphtheria, pertussis and influenza, which can be recommended during pregnancy, live attenuated vaccines like the combined measles-mumps-rubella (MMR) vaccine are contraindicated in pregnant women due to the hypothetical risk of transplacental viral transmission and infection of the fetus (50). However, observations from prenatal MMR immunization administered during the first trimester to women unaware of their pregnancy revealed that the risk for adverse pregnancy outcomes such as spontaneous abortion, hydrocephalus, vaginal bleeding and preterm birth is not significantly increased compared to the general population. Also, fetal infection has not been reported (51).

Resulting from the global rise of vaccine hesitancy, one of the 10 threats to global health (52), transmission of measles is rapidly spreading, which poses a significant hazard to children’s health. Besides common complications related to measles infection in children, such as diarrhea, middle ear infection and pneumonia (53), it has recently been identified that measles can obliterate existing humoral immune memory against a repertoire of pathogens (54, 55). The incomplete reconstitution of the naïve B cell pool and the depletion of previously expanded B memory clones account for this obliteration of immune memory (54). Hence, the susceptibility toward subsequent infections is greatly enhanced after measles infection, which strongly underpins the urgency not only for vaccination of children, but also for women with the intention to become pregnant. This will close a vulnerable gap of neonatal susceptibility toward measles prior to the recommended vaccination at the age of one year and allow to achieve global measles elimination.

In Germany, immunization of adults with MMR is only recommended for individuals with an incomplete or unclear vaccination history (37). Since the age of women at the time of giving birth to their first child has increased by approximately a decade during the last 50 years (56), the window between routine childhood vaccination and onset of pregnancy has also increased. Hence, antibody concentrations might have waned substantially at the time of pregnancy. It has been observed that a MMR vaccination dose administered close to pregnancy induces higher matAB levels in the offspring, irrespective of the total number of vaccine doses given to the mother (57). In countries where pathogens such as measles still circulate within the population and hence, natural infections and boosting through recurrent exposures to the wild-type pathogens are frequent, antibody concentrations are higher compared to those mounted by immunization (57). *Vice versa*, in highly vaccinated populations with low pathogen circulation, antibody concentrations in mothers and their children tend to be lower due to faster decrease of vaccine-induced antibodies and a lack of natural boosting through pathogen exposure (58–60). Gonçalves et al. quantified this observation by measuring anti-measles-IgG in cord blood and found that in infants born to MMR-unvaccinated mothers, who most likely gained their immunity through natural infection, anti-measles-IgG reached 1849 mIU/ml, while cord blood of infants born to MMR-vaccinated mothers only contained 987 mIU/ml of anti-measles-IgG (57). It has been suggested that immune responses toward measles may differ if mounted by natural infection or by vaccination, because different antibody subclasses may be induced and that infants of vaccinated mothers lose passive acquired immunity at an earlier age compared to naturally immune mothers (61).

Studies observing the impact on the neonate in case of rubella reinfection during pregnancy (62–64) have reported that rubella reinfection can occur both in naturally immune women and in women immunized against rubella during childhood. Noteworthy, immunized women are at greater risk for such reinfection, which might be due to differences in the immune response following vaccination or natural infection. The course of rubella reinfection is mostly subclinical, but may have severe consequences such as the congenital rubella syndrome (CRS), though this has been described only in one case (65). Thus, MMR booster doses can be recommended to women planning to become pregnant in order to avoid serious illness of the child if exposed to measles or rubella virus during gestation or during the first months of life.

## Mechanisms of Transferring Immunity to the Newborn: Transplacental Transport of Maternal Antibodies

The wealth of studies summarized so far highlights that maternal antibodies against specific pathogens can be vertically transferred to the fetus and subsequently protect the neonate from infections. Thus, the mechanism of such vertical transfer is a key modulator of neonatal immunity and shall be reviewed in the following.

**FIGURE 2:**
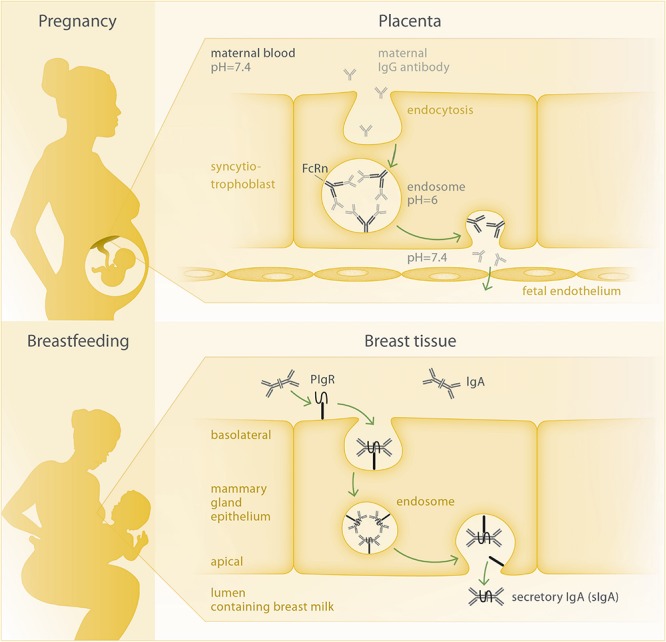
Mechanisms of antibody transfer via placenta and breast tissue. Top: Circulating IgG antibody is taken up into the syncytiotrophoblast cell, where two IgG molecules per FcRn bind at the inner membrane of the acidic endosome. Upon opening of the endosome at the basolateral side of the cell facing the fetal circulation, FcRn releases the IgG molecules due to the increased pH and can then be recycled to perform another transport cycle. Bottom: The joining chain of the dimeric IgA molecule is bound by the polymeric Ig-receptor (pIgR) and both are internalized via endocytosis. At the apical membrane, secretory IgA (sIgA) is being released to the breast milk, as the secretory component of pIgR remains bound to the IgA antibody.

Generally, the placenta poses a barrier which can – at least partially – control and hinder the transmittance of harmful substances from mother to fetus. Hence, a specific and active transport mechanism is needed in order to transfer maternal pathogen-specific antibodies to the fetus. In this respect, the neonatal Fc-receptor (FcRn) plays a key role. It is, amongst other tissues, expressed in placental syncytiotrophoblasts and belongs to the Fcγ receptor family, which characteristically binds the Fc fragment of IgG antibodies and promotes their transport to body sites where specific immunity is needed (66). The IgG binding characteristics of FcRn are highly pH-dependent (67); in acidic environments, FcRn shows a much higher affinity to IgG compared to the physiological pH of 7.4, which is present in maternal and fetal blood. Thus, maternal antibodies are unable to bind to FcRn at the apical side of the syncytiotrophoblasts, which is bathed on maternal blood, but need to be taken up by endocytosis (Figure 2). The amount of antibody that can be transferred to the fetus depends on the amount of FcRn expressed by syncytiotrophoblasts. If all FcRn are engaged in IgG transport, additional IgG molecules will be degraded in the lysosome, as they are not receptor-bound. Thus, antibody transfer is a saturable process and will stagnate once maternal antibody concentrations reach a certain level, which has been defined as a total IgG of 15 g/L (68). Transplacental IgG transport starts early in gestation (10, 69), though still at low efficacy. With the continuation of pregnancy, FcRn expression and transplacental transport increase, peaking during the last four weeks of gestation (9). It is tempting to speculate that the increased cell mass of the growing placenta accounts for the mere increase in FcRn and related higher antibody transport rate.

To date, research on factors influencing the FcRn expression is scarce. In one study, the effect of vitamin A supplementation during pregnancy on the immune response following maternal influenza vaccination during pregnancy has been assessed. Here, a possible influence of vitamin A on FcRn expression has been proposed, but this aspect is still highly speculative and data are based on a small sample size (70). Data on sex-specific differences in placental FcRn expression is also currently lacking.

Interestingly, not all IgG subclasses are equally transferred, as FcRn mainly transports IgG1, with decreasing efficacy for IgG4, IgG3, and IgG2 (69). Structurally different antigens have been shown to induce different IgG subclasses and thus, are transferred in varying amounts. While protein antigens, such as pertussis toxin and pertactin, tetanus toxin or the measles virus elicit IgG1, polysaccharide antigens, as found on the surfaces of bacteria like Haemophilus influenzae type b or Neisseria meningitidis, induce IgG2 (2). Since the latter subclass is being transported less efficiently, newborns might lack specific immunity toward pathogens which mainly present polysaccharide antigenic structures, such as most bacteria.

The most predictive factor of transplacental antibody transfer is the level of maternal antibody (10). Higher gestational age, recent maternal vaccinations, a balanced maternal nutritional status and male gender of the newborn have been shown to positively influence maternal antibody concentrations in the infant (1, 71, 72).

Observations dating back some decades indicate that apart from IgG being transported across the placenta as a single molecule, it can also be transported as an IgG-immune complex (IgG-IC) involving IgG and it’s respective antigen (73). In this study, serum tetanus antigen reached nearly the same levels in mother and infant at birth, suggesting an active transfer of anti-tetanus IgG-IC. Active transfer could not be observed for different pregnancy-related proteins such as alpha-fetoprotein (AFP) and human chorionic gonadotropin (hCG), as their concentrations highly differed between mother and child, indicating a transmission by low-rate diffusion. More recently, influenza-specific fetal IgM could be detected in cord blood upon maternal influenza vaccination during pregnancy, suggesting that anti-influenza IgG-IC had been transferred to the fetus, followed by a fetal B- and T-cell immune response against influenza elicited by the IC *in utero* (74). Together with the observation that IgE, which plays a major role in allergy pathogenesis, can also be transported via the placenta as an IgG-IgE-IC (75), these findings have a great impact on understanding neonatal immunity and the development of atopy in children.

## Mechanisms of Transferring Immunity to the Newborn: Transfer of Maternal Antibodies Via Breastmilk

Another substantial element of neonatal immunity is the intake of breast milk, which contains a significant amount of secretory IgA. Also, maternal immune cells, such as IgG-producing memory B cells and CD4^+^ T cells, can be detected in breast milk (9, 76).

The dimeric IgA antibodies are produced by plasma cells in the mammary gland as well as in other tissues associated with mucosal surfaces. The epithelial cells of the mammary acini transport the IgA molecules from the connective tissue to the breast milk via transcytosis, involving the polymeric Ig receptor (pIgR) (77, 78) (Figure 2). The two IgA subclasses present in humans, IgA1 and IgA2, are distributed differently along mucosal membranes, with IgA1 being mainly present in the respiratory tract, saliva, serum and skin and IgA2 being the main secretory antibody of the intestine (79). In their seminal review, Hanson and Winberg already concluded that breast milk IgA is not absorbed by the infant’s gut, but rather coats the mucosal surface of the intestine to protect it from pathogens (80).

Several studies have unveiled that the consumption of breast milk by the neonate is beneficial to its health (Table 1). One example is the enhanced transfer of influenza-specific and neutralizing IgA to the neonate upon influenza vaccination of the mother during pregnancy (81), which was associated with a decreased number of respiratory illness of the infants during the first six months of life. Whether this effect results from the increased amount of specific breast milk IgA or from the transplacental transfer of maternal influenza-specific IgG remains to be elucidated. Cross-fostering may provide an answer and considering the growing number of milk banks, such studies may become feasible.

Another study recently highlighted a breast milk IgA-mediated protection from necrotizing enterocolitis (NEC) in preterm infants (82). The pathogenesis of NEC seems to be mainly driven by an altered sIgA binding pattern of intestinal bacteria in the newborn, since the proportion of IgA-bound bacteria was much lower in infants developing NEC compared to healthy newborns. Formula-fed infants were more likely to develop NEC than breastfed infants, presumably due to the absence of maternal IgA in formula alimentum.

Moreover, in women with respiratory tract infections during pregnancy, the proportion of IgA1 in colostrum was higher, while in women with gastrointestinal infections, levels of IgA2 were increased (83). These observations suggest that the mother’s immune system seeks to shield the infant from the specific pathogens of the surrounding environment. Similarly, as shown in basic science models, maternal antibodies can potentially retain microbial molecules and transmit them to the offspring via the placenta and breastfeeding. Subsequently, the offspring are able to avert an inflammatory response to microbial molecules and allow colonization of intestinal microbes (84).

Overall, the beneficial effect of breastfeeding for infant’s health seems to affect various mucosal membranes, such as the respiratory and gastrointestinal tract, hereby protecting the neonate from infections. Thus, the current recommendations of the WHO to exclusively breastfeed an infant during its first 6 months of life (85) may indeed provide optimal starting conditions for the child’s postnatal immunity.

## Mechanisms of Transferring Immunity to the Newborn: Maternal Microchimerism

Besides antibody-mediated immunity transferred during pregnancy, it is also conceivable that pathogen-specific maternal immune cells migrate to the unborn child. It is well known that maternal immune cells can be transferred to the fetus via the placenta (86), and also via breast milk (76). These cells can then remain in the offspring until adulthood, as shown among lymphoid and myeloid compartments of peripheral blood in healthy adult women (87). Due to the low frequency of these cells in the offspring, they are referred to as maternal microchimeric cells and a considerable percentage of such cells are T cells, which can be retained for a long period of time (88).

In general, upon infection, pathogen-specific CD8^+^ T cells remain in peripheral tissues and act as sentinels. Upon antigen re-encounter, they rapidly produce inflammatory cytokines and thereby induce a state of alertness in the local environment and recruit inflammatory cells. Thereby, a small number of pathogen-specific T cells can provoke a fast and fulminant response (89, 90). Interestingly, in the context of pregnancy, there is direct evidence for such transfer of protective maternal T cells. In a human infant with severe combined immunodeficiency suffering from Epstein-Barr virus (EBV) infection, large numbers of maternal CD8^+^ T cells could be detected. These cells were phenotypically activated and secreted IFN-γ in response to EBV antigen. Other hematopoietic cells were of offspring genotype, indicating that the CD8^+^ T cells originated from mature maternal T cells and not form transferred hematopoietic stem cells (91). Moreover, high frequencies of pre-existing effector CD8^+^ T cells directed against conserved core protein epitopes of influenza virus strains correlate with a milder course of influenza infection caused by other influenza virus strains, thus providing strong evidence for a cross protective function of memory CD8^+^ T cells against heterologous influenza strains (92). Based on these empirical evidences, it is appealing to speculate that pathogen-specific maternal microchimeric T cells also convey passive cellular immunity to the offspring.

## The Downside of Neonatal Passive Immunity: Maternal Antibodies Interfere With the Infant’s Response to Vaccination

Despite the significant health benefits resulting from maternal vaccination during pregnancy for mother and infant, there is also a downside to it. Many studies have demonstrated that high levels of maternal antibodies in the infant hamper the immune response required to mount humoral immunity upon routine childhood vaccinations (68). This inhibitory effect of maternal antibodies on the antibody generation by the infant’s immune system, which is commonly referred to as “blunting,” can affect neonatal immunity for up to more than one year of age, depending on the level of maternal antibodies in the neonate at birth. Interestingly, blunting occurs irrespective of the type of vaccine applied, including measles, influenza and pertussis vaccines (68).

**FIGURE 3:**
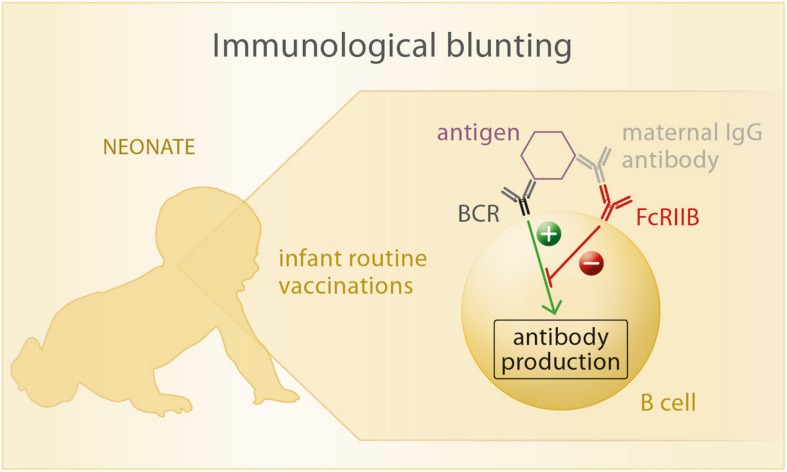
Upon exposure of the neonate to vaccine antigens, the antigen is recognized by its specific B cell receptor (BCR). If maternal antibodies are present in the child’s circulation, they bind to the vaccine antigen as well as to the Fc-receptor FcRIIB that is also expressed on B cells. Thus, a cross-link between BCR and FcRIIB is formed, which inhibits antibody production of the B cell in response to antigen recognition.

The most common explanation for blunting involves a cross-link between the B cell receptor (BCR) and the Fcγ receptor FcγRIIB (68, 93), both expressed on the surface of B cells. Each BCR has a unique affinity to a certain pathogen epitope, which can also be recognized by specific maternal antibodies. These again can be ligated to the FcγRIIB by their Fc fragment. When the infant is being vaccinated, pathogen fragments enter its circulation and can be bound both by BCR and maternal antibodies at the same time, which leads to contradicting signals within the B cell. While the BCR recognizes the new antigen and emanates signals leading to plasma cell differentiation and antibody production, FcγRIIB signalizes the presence of specific antibody to this particular antigen and inhibits further antibody production. In consequence, the stimulatory BCR signal is being inhibited and no antibody production can be initiated by the infant’s immune system (Figure 3).

Very recently, a large study has thoroughly addressed the topic of blunting by maternal antibodies (94). Here, children’s antibody responses to routine early life vaccinations against Hepatitis B, tetanus, diphtheria, pertussis, polio, pneumococcus, rotavirus, MMR, and meningococcus have been associated with maternal vaccine responses using inactivated influenza vaccine or Tdap during pregnancy. While maternal influenza vaccination did not affect the infant’s vaccine responses, maternal immunization with Tdap resulted in significantly lower vaccine responses to specific (diphtheria and pertussis) and heterologous antigens (polio and pneumococcus) in the child. This observation has sparked the notion that maternal antibodies present in the neonate bind to the diphtheria-toxin derived carrier protein of the pneumococcal vaccine before the neonatal host can mount an immune response against the heterologous antigens bound to the carrier protein. Also, reduced blunting has been described upon infant immunization with acellular as compared to the whole cell pertussis vaccine (19). In order to support maternal vaccination strategies, the consequence of Tdap-booster immunization at 13 or 15 months of age upon maternal Tdap vaccination during pregnancy has been assessed. Here, Tdap booster doses overcame an initially observed blunting effect caused by high maternal antibody levels (95, 96).

Approaches seeking to bypass the process of blunting are nowadays tested, such as alternative vaccination routes and the simultaneous injection of antigen-specific IgM or agents that stimulate the production of interferon-α along with the vaccine (68). These adjuvants have been suggested to counteract the inhibitory signal produced by FcγRIIB, thus leading to B cell activation and antibody production following immunization even in the presence of maternal antibodies. Additionally, as reported by studies dating back two decades and more, maternal antibodies do not interfere with T cell priming of the infant (97–99). These observations support that sufficient protection can still be reached at the time when maternal antibodies have completely waned in the infant at an age of approximately 6 months, even if the first vaccination did not trigger a significant humoral immune response.

There is still ambiguity regarding the occurrence of a blunting effect, as it was not confirmed in all studies assessing it (22). Hence, future studies are required to confirm the underlying mechanisms of blunting and T cell priming in order to ensure highest efficacy of neonatal immunization. Clearly, blunting of vaccine responses in infants might increase the susceptibility to certain early life infections. Considering the advantages related to maternally derived passive immunity for the neonate, blunting however, has been described as an acceptable trade-off (94).

Noteworthy, a number of articles published in the 1980s support that anti-idiotypic antibodies are also transferred from the mother to the fetus via the placenta (100) and by breast milk. Anti-idiotypic antibodies are directed against molecular patterns (idiotypes) located close to the antigen-binding site of pathogen-specific antibodies and are being elicited as part of the regular immune response. A proportion of anti-idiotypic antibodies carry an “internal image” of the antigen for which their idiotype antibodies are specific. Thus, anti-idiotypic antibodies are thought to stimulate B-cells in an antigen-independent manner and subsequently lead to the production of antigen-specific, idiotype-carrying antibodies with neutralizing ability (101–104). Low levels of maternally derived anti-idiotypic antibodies have been shown to provide a significant priming effect on the immune system of the neonate, protecting neonatal mice from pathogen challenges (104). Conversely, a high dose of maternally derived idiotype and anti-idiotypic antibodies, acquired via transplacental transfer or breastfeeding, may yield to the observed blunting effect. Hence, based on the immune network theory by Jerne (105), complex regulatory mechanisms involving idiotype and anti-idiotypic antibodies underlie the infant’s vaccine responses. Strikingly, using monoclonal anti-idiotypic antibodies as a vaccine to immunize against measles, mumps and rubella, against which to date can only be vaccinated later in life, could allow to induce protection already at birth. This would overcome a major window of vulnerability and reduce the burden of disease in young infants.

## Vaccination Compliance During Pregnancy

Despite all these evidences highlighting the benefit of vaccinations during pregnancy for mother and child, poor vaccination compliance among women during their reproductive years is still an alarming clinical problem. This poor vaccination compliance is the result of a number of factors, including the neglect of healthcare providers to offer vaccination, limited availability and high costs of vaccines, doubts of the effectiveness of vaccinations, concerns about the safety of the vaccine for mother and fetus (106, 107). Continuous accumulation of evidence that vaccination strategies can yield to significant health advantages for mother and child and the communication to researchers, lay individuals and stake holders will hopefully improve the vaccination compliance in the near future.

## Conclusion

A wealth of published evidence strongly underpins that vaccination during pregnancy is advantageous not only for maternal health, but also for children’s well-being. Especially maternal vaccination against tetanus, diphtheria, pertussis and influenza has been convincingly demonstrated by a large number of studies to be safe, immunogenic and to provide significant immunity to the newborn. The latter could not only be confirmed by the mere presence of maternally derived pathogen-specific antibodies in newborns, but indeed a reduced risk for pertussis and a broad protection from lower respiratory tract infections, even beyond infection with the influenza virus. Noteworthy, the downside of high levels of maternal antibodies against pathogens, the induction of immunological blunting in the infant, seems to dampen the neonatal response to early life vaccinations and causes a threat to neonatal health. The reduced risk for neonatal infections due to maternally derived immunity however, clearly proves that blunting-related disadvantages are outweighed by the advantages. This has been confirmed by a recent study which reports that measles vaccination of infants in the presence of maternal anti-measles antibody significantly reduced overall infant mortality, compared to vaccinated infants without maternal antibodies (108). Lastly, the poor vaccination compliance is a challenge that must urgently be met, for example by implementing maternal immunization platforms through which education and communication of vaccination-related benefits are facilitated and vaccines are routinely offered in order to increase the willingness and subsequently the vaccination rate of pregnant women.

## Author Contributions

MA and PA jointly outlined the article, developed the figures, and wrote the review article.

## Conflict of Interest

The authors declare that the research was conducted in the absence of any commercial or financial relationships that could be construed as a potential conflict of interest.
